# Effect of graphite and graphene oxide on thorium carbide microstructural and thermal properties

**DOI:** 10.1038/s41598-021-87621-0

**Published:** 2021-04-27

**Authors:** S. Corradetti, S. M. Carturan, M. Ballan, R. Eloirdi, P. Amador Celdran, O. Walter, D. Staicu, O. Dieste Blanco, A. Andrighetto, L. Biasetto

**Affiliations:** 1 INFN-Laboratori Nazionali di Legnaro, Viale dell’Università 2, 35020 Legnaro, PD Italy; 2 Dipartimento di Fisica e Astronomia, Università di Padova, Via Marzolo 8, 5131 Padua, Italy; 3 Joint Research Centre, Directorate G for Nuclear Safety and Security, European Commission, Postfach 2340, 76215 Karlsruhe, Germany; 4 Dipartimento di Tecnica e Gestione dei Sistemi Industriali, Università Di Padova, Stradella San Nicola 3, 36100 Vicenza, Italy

**Keywords:** Materials science, Nanoscience and technology, Physics

## Abstract

Thorium carbide to be tested as target material for the production of ^225^Ac with the ISOL method, was produced via carbothermal reduction of ThO_2_ nanoparticles by graphite and graphene oxide, respectively. The use of graphene oxide (GO) as carbon source resulted in a reduced reactivity compared to graphite, confirmed by the presence of unreacted ThO_2_ mainly in the core of the samples. The reacted ThO_2_ or ThC_2_–GO showed a faster reactivity in air, mainly observed as ThC_2_ amorphization. The specific surface area of the ThC_2_–GO samples was almost doubled compared to ThC_2_–graphite samples. The effect of these microstructural features was analysed in terms of thermal diffusivity and calculated thermal conductivity that were both reduced in ThC_2_–GO samples, however the difference with ThC_2_–graphite samples decreased at increasing temperature. The present study shows that the use of unreduced GO inhibits the solid-state reaction between ThO_2_ and C; on the other hand, the high reactivity of the ThC_2_ so produced is expected to be beneficial for the ^225^Ac production with the ISOL method, affording a high release efficiency. It is expected that the use of reduced GO could represent a good solution for highly efficient ThC_2_ targets.

## Introduction

Since its appearance in clinical research literature in the late 1990’s, Targeted Alpha Therapy (TAT) has been considered an extremely promising approach to treat cancer^1^. The benefits of using alpha-emitting radioisotopes to selectively induce localized damage to cancer cells come from peculiar properties of alpha particles with respect to beta radiation. Due to their much larger mass, alpha particles result in a higher linear energy transfer, i.e., they deposit higher amounts of energy over shorter ranges.

Among the potentially employable alpha-emitting radioisotope for this purpose, ^225^Ac has recently attracted much interest due to the combination of a suitable 10-day half-life, the emission of four alpha particles in its decay chain and the generation of ^213^Bi, another isotope considered for TAT applications^2^. The development of ^225^Ac-based radiopharmaceuticals presents several challenges, mostly linked to the availability of sources and/or production techniques of this particular isotope and to the purity of the obtained products. At present, ^225^Ac for clinical research is primarily produced from the decay of ^229^Th stocks located in the USA, Russia and Germany, in turn obtained from stockpiles of ^233^U^3^. Other explored approaches are the use of ^226^Ra targets bombarded by protons or neutrons^4^ and the direct irradiation of ^232^Th with high-energy protons^5^. None of these techniques is free from the necessity of developing several stages of chemical purification to eliminate co-produced and undesired isotopes of actinium and other elements.

With the aim of developing new and more efficient production methods, ISOL (Isotope Separation On-Line) facilities are being considered as sources to get high purity ^225^Ac for research purposes^2^. In the framework of such existing or future facilities, traditionally devoted to the production of radioactive ion beams for nuclear physics, several research programs on medical isotopes production are now ongoing. Examples include MEDICIS at CERN^6^, ISAC at TRIUMF^7^, ISOLPHARM at INFN^8^ and ISOL@MYRRHA at SKC-CEN^9^. The presence of a mass separation stage in these infrastructures ensures high purity, due to the absence of same-element contaminants, which cannot be removed with chemical separation methods.

The research reported here is aimed at the development of a nanostructured thorium carbide-based target for ^225^Ac production with the ISOL method. Historically, thorium carbide targets have played a rather marginal role in ISOL facilities^10,11^ compared to the commonly used uranium carbide ones, due to lower yields of radionuclides relevant for nuclear physics and more difficult synthesis and handling. The need for optimized targets at the nanoscale level has lately arisen in the ISOL target community; stable porous nanostructures with high specific surface area (SSA) have been associated with increased performance consistency during irradiation, guaranteeing high yields to experimental users^12,13^.

Our synthesis of high-SSA thorium carbides, was inspired by recent work carried out at JRC Karlsruhe on thorium oxide nanopowders^14,15^. The idea behind this work was to let nano-ThO_2_ react with two different carbon sources (graphite and graphene oxide) at high temperature in an inert atmosphere, to obtain ThC_2_-carbon nanocomposites and to verify the retention of high SSA in the final material. Composites materials demonstrated^16^ better thermal properties and thus are of interest for our application. In our previous collaborative work, graphene had the effect of improving thermal properties of uranium carbide–carbon composites^17^. In the present study, graphene oxide was used, with the aim of exploiting its low-temperature reduction^18^ and the related gas release during thermal treatment, thought to be beneficial for the creation of interconnected micro- and meso-porosity, and consequently for obtaining a high SSA.

## Results

An overview of the properties of graphite and graphene oxide–derived thorium carbide pellets is given in Table 1. In the following, these materials will be referred to as ThCx–graphite and ThCx–GO, respectively. The results of elemental analysis (carbon and oxygen content) on both the starting materials and the final pellets are shown in Table 2. Both materials showed higher weight losses with respect to those calculated based on stoichiometry (17 wt%), as reported in “Methods” section. This is generally attributed to phenomena such as water adsorption on starting reagents, binder decomposition and oxygen contamination in the carbon sources, none of which are considered in the stoichiometry. On the other hand, if one considers the actual C/O ratio found in ThCx–graphite and ThCx–GO by elemental analysis, the weight loss calculated with the corrected stoichiometry is around 20 ± 1 wt%, this agrees with the experimental values of 19.6 ± 0.1 wt% for ThCx–graphite but is higher than 17.7 ± 1.6 wt% for ThCx–GO. The remaining graphite (1.4 wt%) or GO (3.3 wt%) is then transformed to the C being present in the sample. The lower weight loss observed for ThCx–GO indicates incomplete conversion, which could be an effect of increased surfaces or lower reactivity, as shown in the following. Despite showing different volumetric shrinkages and weight losses, the two materials ended up having similar densities and, thus, total porosities. The total porosity was obtained from a calculation which considers the designed stoichiometry of the final materials (ThC_2_ + 2C), and therefore does not take into account any discrepancy in the composition of the final products. The data reported in Table 2 are close to those reported by the supplier’s certificate of analysis. Graphite has a higher C content than graphene oxide, in which O is present together with H either as water or in different organic groups. In the case of ThCx–graphite and ThCx–GO, again considering the final anticipated stoichiometry ThC_2_ + 2C, Th represents about 83 wt% of the final mass. The three ThCx–GO samples used for the elemental analysis showed a difference in the carbon content indicating a clear non-homogeneity in the sample, resulting in a higher error with respect to ThCx–graphite.

**Table 1:** Composition and properties of the samples.

Sample	Composition before thermal treatment (wt%)				Properties after thermal treatment (T = 1923 K, Argon flow)			
	ThO_2_	Graphite	Graphene oxide	Phenolic resin	Density (g/cm)	Volumetric shrinkage (%)	Weight loss (wt%)	Total porosity (vol%)
ThCx–graphite	77.1	21.0		1.9	4.0 ± 0.2	28.1 ± 4.1	19.6 ± 0.1	43.0 ± 1.6
ThCx–GO	77.1		21.0	1.9	4.0 ± 0.2	23.2 ± 0.12	17.7 ± 1.6	43.1 ± 1.6

**Table 2:** Carbon and oxygen concentrations obtained by elemental analysis and provided by the supplier.

Sample	Elemental analysis		Supplier data	
	C content (wt%)	O content (wt%)	C content (wt%)	O/H content (wt%)
Graphite	95.9 ± 1.8	0.6 ± 0.1	99.99	−/−
Graphene oxide	89.8 ± 3.1	5.5 ± 0.5	83.4	8.1/4.5
ThCx–graphite	19.1 ± 0.2	2.6 ± 0.2	–	–
ThCx–GO	19.7 ± 1.0	3.8 ± 0.7	–	–

**Figure 1:**
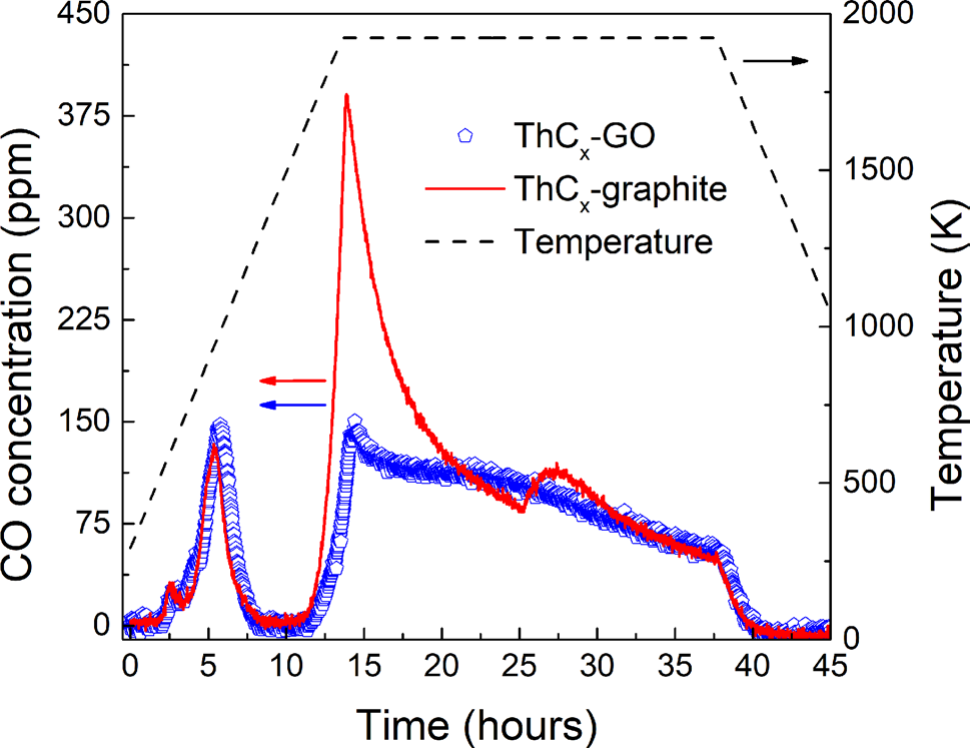
Gas evolution during the two thermal treatments. Symbols are used to represent data for ThCx–GO whereas only for the sake of clarity a line is used for ThCx–graphite.

Figure 1 shows the evolution of the CO release during the separate thermal treatments of ThCx–graphite and ThCx–GO samples. Since two batches of roughly the same mass of reagents were loaded into the furnace in each treatment (6 pellets with a total mass of about 3 g), the evolution and total quantity of emitted CO should be similar. While this is true for the low-temperature CO release associated with binder decomposition^19^, a remarkable difference can be seen at high temperature. Above 1700 K indeed a higher gas release was observed in the ThCx–graphite treatment. Part of it could be attributed to the oxidation of graphite components of the furnace, since the thermal treatment of the ThCx–graphite was carried out before that of the ThCx–GO and after a period of inactivity. However, a further heat treatment of the samples reproduced the characteristic gas release and suggested that the outgassing of the internal furnace wall is minor. This outgassing usually happens at temperatures well below 1700 K, so the difference in CO release can be better ascribed to incomplete carburization of ThCx–GO.

**Figure 2:**
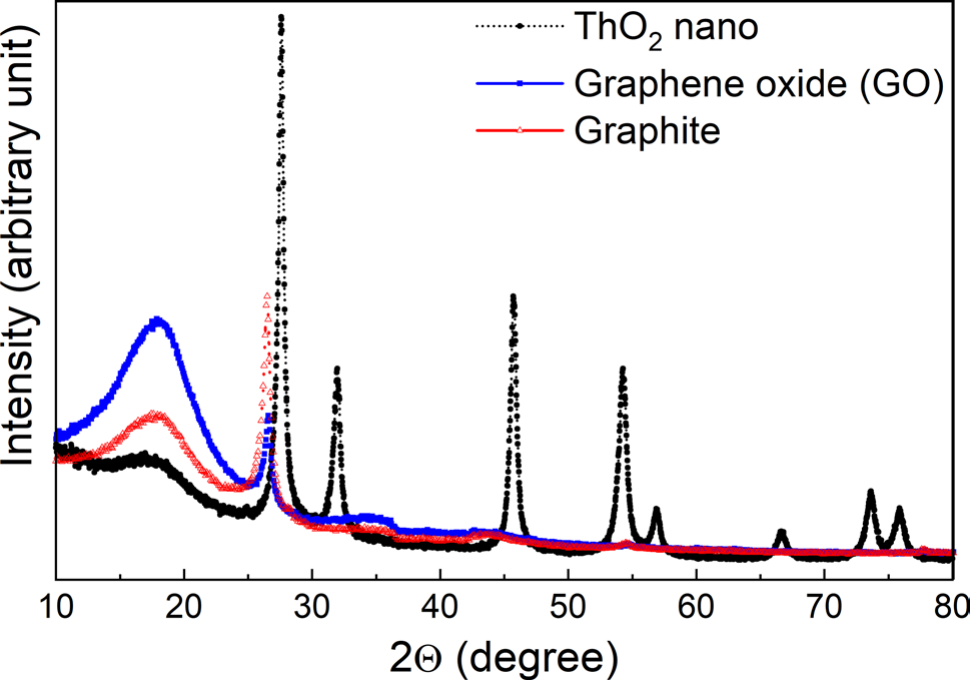
XRD patterns of starting materials.

**Figure 3:**
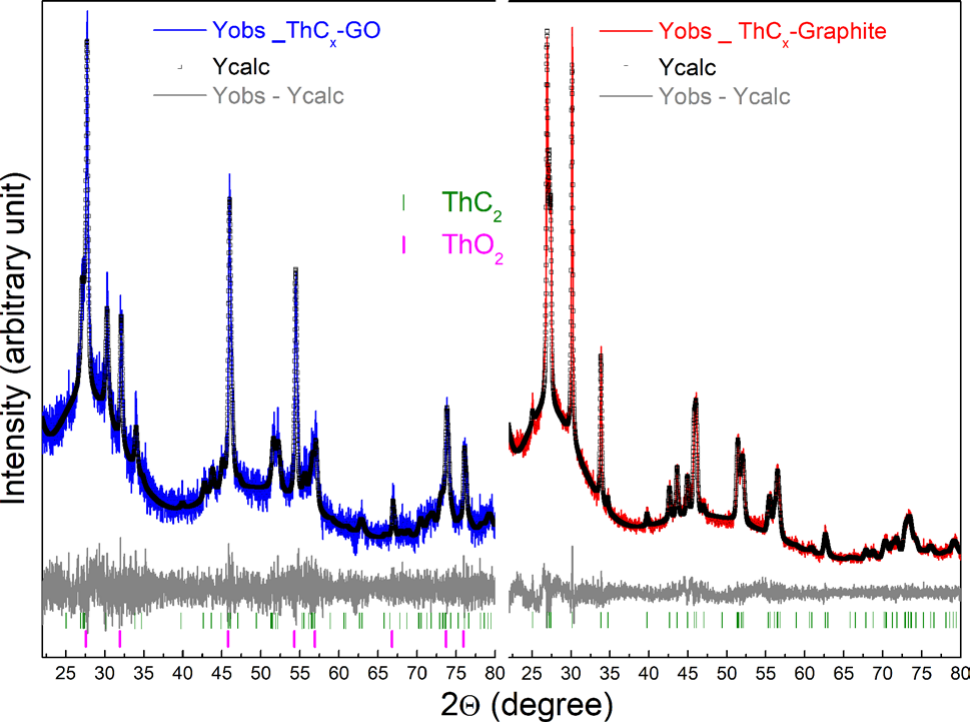
XRD patterns of ThCx–GO and ThCx–graphite. Y_obs are experimental data, Y calc are calculated data from the Lebail fitting.

Figure 2 reports X-ray diffraction (XRD) patterns of the starting reagents, whereas those of ThCx–graphite and ThCx–GO are shown in Fig. 3. The ThO_2_ nanoparticles obtained by hydrothermal decomposition of the thorium oxalate have a size of about 7 (± 1) nm routinely verified by powder XRD and calculated from the line broadening of 5 isolated peaks in the diffractogram. They have been analysed by TEM, as reported in^14,15^. Figure 3 clearly shows that ThCx–graphite is solely composed of ThC_2_ as the crystalline phase whereas ThCx–GO contains a mixture of two crystalline phases ThC_2_ and ThO_2_. The analyses of the XRD patterns of both samples including the Le Bail fitting confirm the cubic structure for ThO_2_ (a = 5.5988 (2) Å, Fm-3m_space group 225) and the monoclinic structure for ThC_2_ (a = 6.694 (2) Å, b = 4.2405 (12) Å, c = 6.746 (3) Å, β = 103.84 (2)°, C2/c-space group 15) found for the ThCx–GO whereas for the ThCx–graphite only the monoclinic structure is found for ThC_2_ (a = 6.6983 (6) Å, b = 4.2369 (4) Å, c = 6.7478 (6) Å, β = 103.836 (3)°, C2/c-space group 15). This could arise either from an incomplete reaction, as suggested by the aforementioned CO release during the treatment and the lower mass loss during the thermal treatment, or with a lower probability from oxidation during the handling and transport of the samples to the characterization devices.

**Figure 4:**
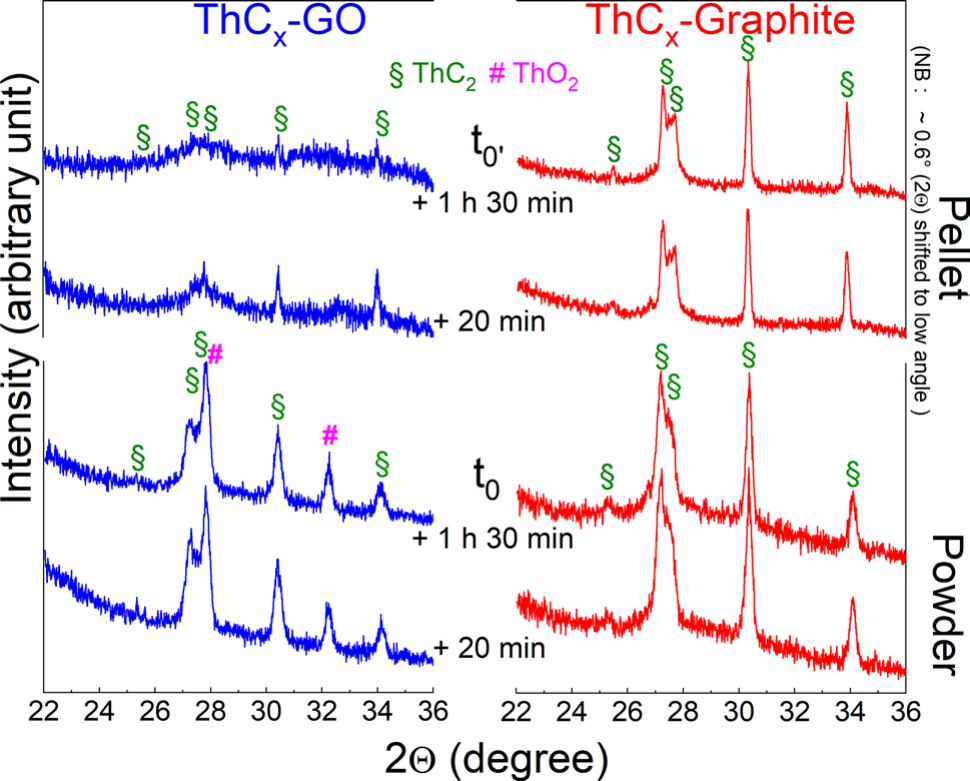
XRD patterns of ThCx–GO and ThCx–graphite pellets (top) and crushed powders (bottom) after embedding in oil and measurement in air.

To investigate this phenomenon of possible ThC_x_ oxidation in more detail, XRD patterns were collected at different times with samples embedded in oil and measurements under air on both the pellet surfaces and the crushed powders, as shown in Fig. 4. Time t_0_ corresponds to the starting of the XRD measurement. The results unambiguously show that:

- ThCx–graphite is more stable both on the pellet surface and the crushed powder, as indicated by the main ThC_2_ peaks, which remain unaltered even after prolonged exposure in air, embedded in oil.
- The surface of ThCx–GO pellets (left, top, Fig. 4) contains ThC_2_, peaks loose intensity over time indicating amorphization.
- Oxygen in form of ThO_2_ is present in the entire ThCx–GO sample (crushed powder) from the beginning. It is thus not possible to assign its appearance to the partial hydrolysis or oxidation of carbide resulting from exposure to moist air. Therefore, it must be residual (unreacted) in accordance with reduced weight loss as reported in Table 1, which indicates incomplete conversion.

**Figure 5:**
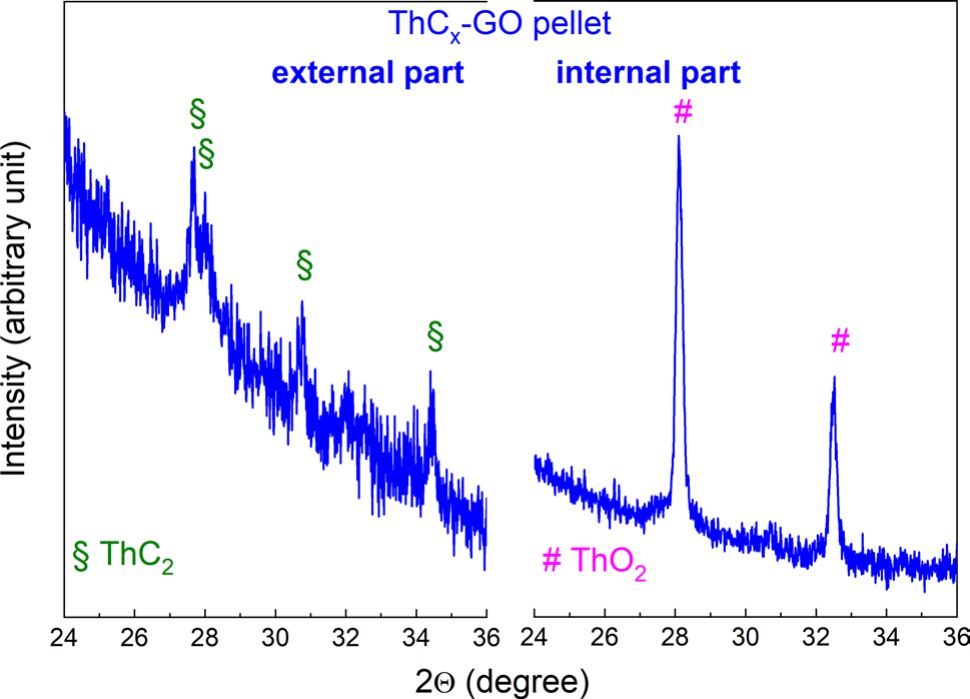
XRD patterns of ThCx–GO external and inner surfaces.

In order to shed more light on the ThCx–GO composition, a carburized sample was studied in more detail (Fig. 5) by polishing it until half thickness was reached and taking an XRD spectrum of the inside surface, to be then compared to the outside surface. The results agree with those of Fig. 4: oxygen is found inside the pellet and not on the surface, providing additional evidence that the oxide–carbide conversion was incomplete. In fact, the inside surface seems to be composed only of ThO_2_, even though the presence of ThC_2_ cannot be excluded completely from the diffractogram (Fig. 5), since the ThC_2_ XRD peaks are less intense than the ones for ThO_2_ due to the lower symmetry (monoclinic for ThC_2_ vs cubic for ThO_2_).

Raman spectra of graphite, GO, ThCx–graphite and ThCx–GO samples have been collected and are reported in the Supplementary Data S1 section. The main features of graphitic carbon-based materials, G and D bands at 1500 and 1350 cm^−1^^20^, are observed. The most intense peak for the graphite is the G band at 1500 cm^−1^, while for GO, D-band and G-band have equal intensity. The G peak is ascribed to the bond stretching of all pairs of sp^2^ atoms in both the rings and chains, while the D band is ascribed to defects in the sample and its intensity is related to the degree of disorder. Considering these assignments and comparing the samples after carburization, ThCx–GO shows the highest ratio of intensity I_D_/I_G_ thus pointing to the highest amount of defects^19^.

**Figure 6:**
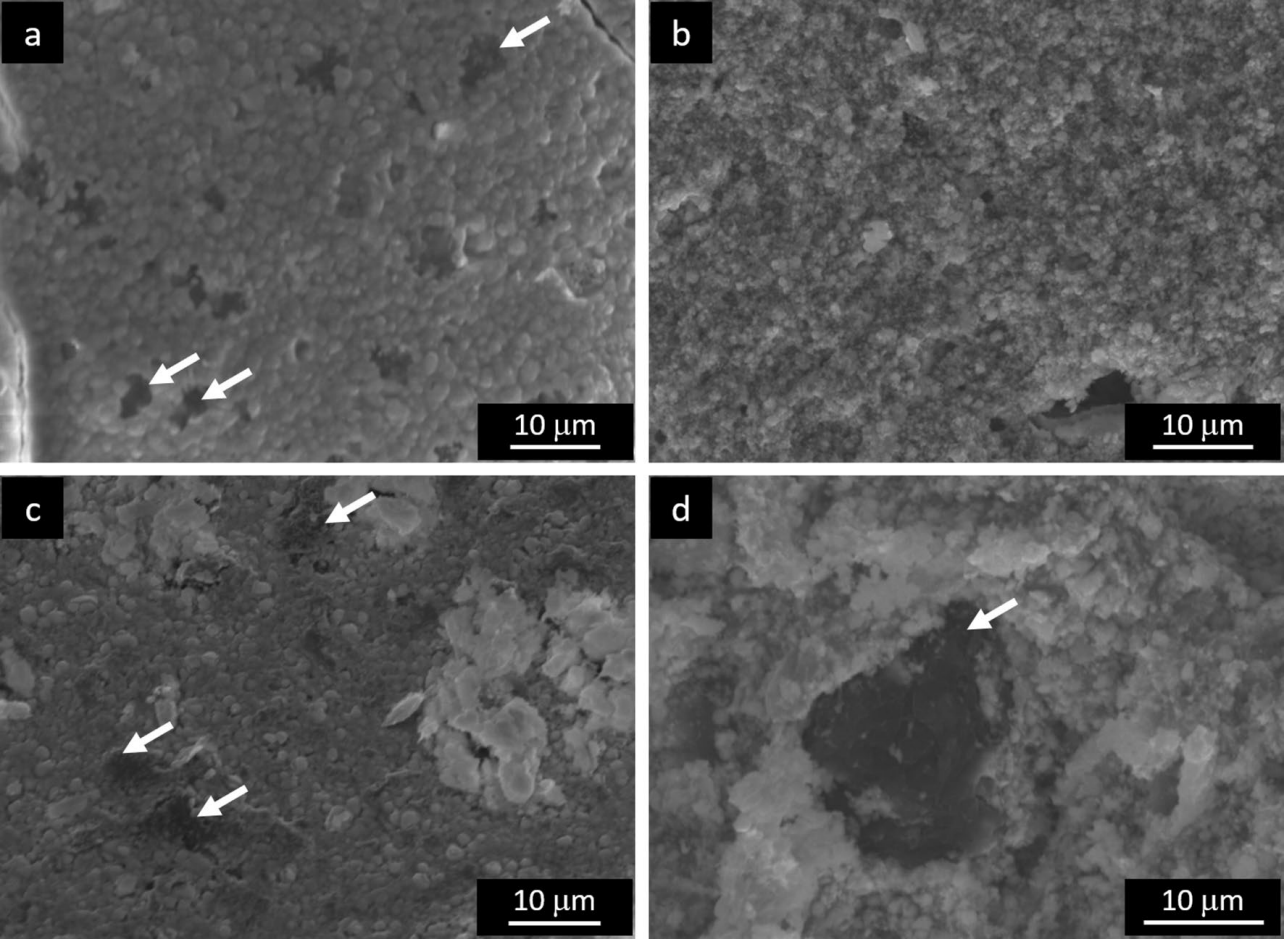
SEM images taken on the surface and along thickness of (**a**,**b**) ThCx–GO and (**c**,**d**) ThCx–graphite. White arrows highlight residual carbon.

SEM images taken on the surface of samples of the two materials are reported in Fig. 6. Both show a microstructure composed of a dispersion of residual carbon (dark grains) in a partially sintered thorium carbide matrix. In ThCx–GO, carbon is clearly visible, contrary to what was found in UC_x_–graphene in another study^17^, but it is equally well dispersed. It must be remarked that in this case graphene oxide was used instead of pure graphene. ThCx–graphite microstructure resembles that of previously obtained carbides using the very same graphite^17,19^, whose size is in the order of tens of μm.

**Figure 7:**
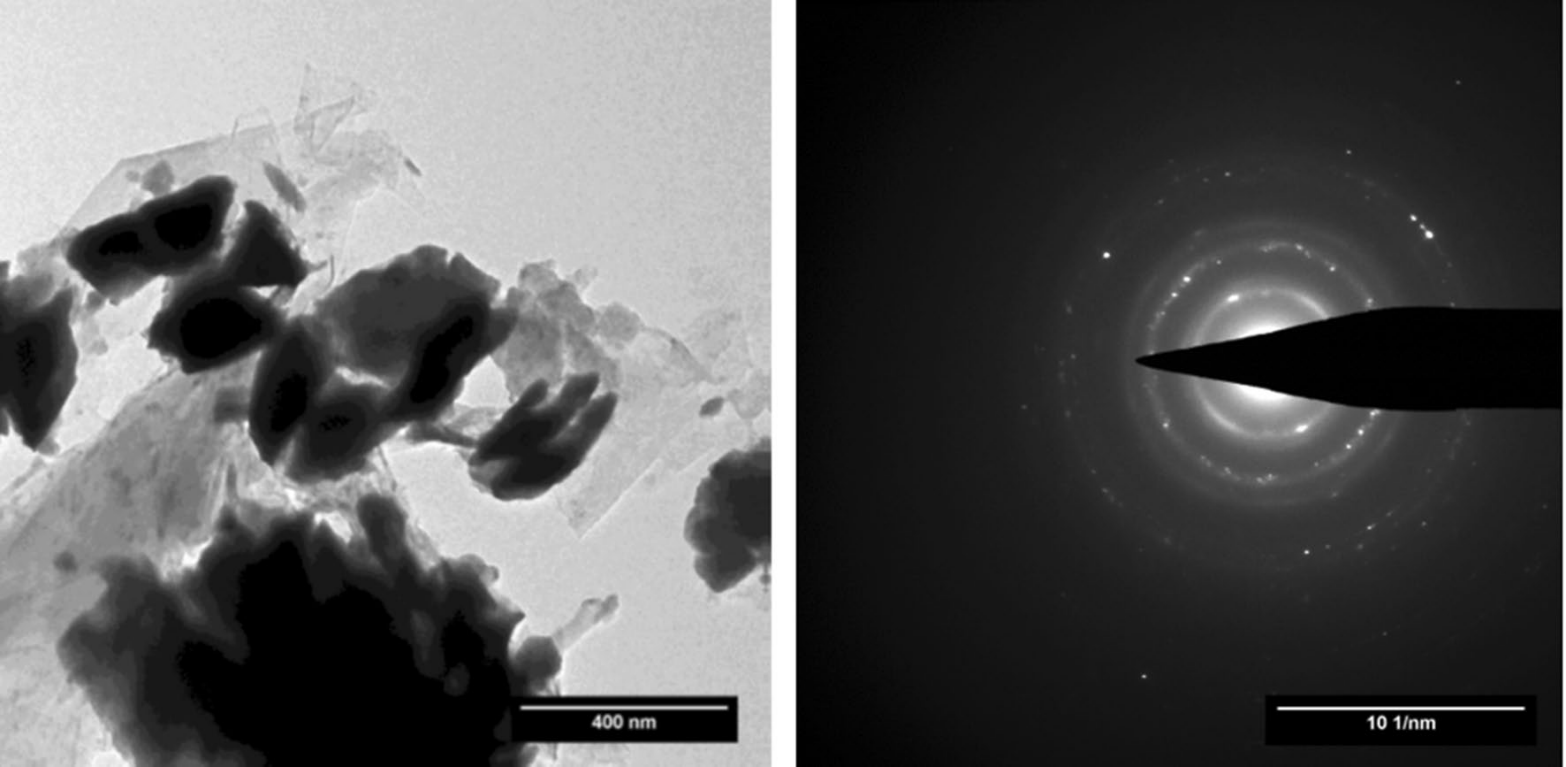
TEM image and ring diffraction pattern taken on ThCx–GO.

**Figure 8:**
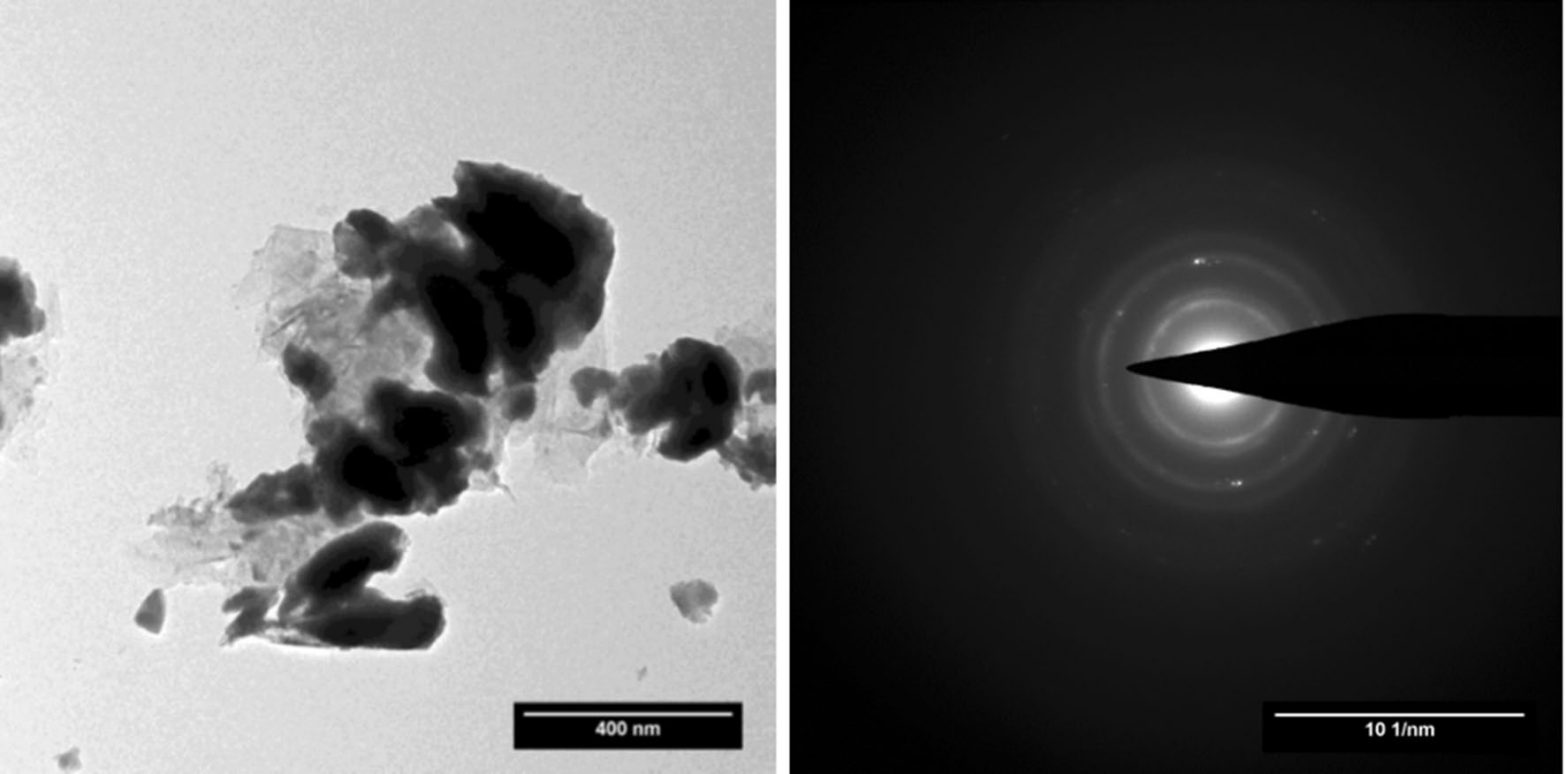
TEM image and ring diffraction pattern taken on ThC_x_–graphite.

TEM images and the corresponding ring diffraction patterns are reported in Figs. 7 and 8 for ThCx–GO and ThCx–graphite, respectively. As in SEM, not many structural differences can be observed between the two materials. No evidence of a discrepancy in carbon distribution around thorium carbide grains is found, contrary to what reported for uranium carbide^17^. The analysis of ring diffraction patterns suggests, however, that the crystalline part of the ThCx–GO sample is polyphasic, with the onset of a cubic phase (ThO_2_) and a monoclinic one (ThC_2_), whereas ThCx–graphite is found to be monophasic.

N_2_ physisorption analyses have been performed on the starting materials, ThO_2_ nanoparticles, graphene oxide and graphite, and on the composite pellets after the carburization reaction. While the analysis on GO and graphite was carried out under standard conditions using a Micromeritics ASAP-2020 surface area and porosimetry system, measurements on ThO_2_ powders and on samples after thermal treatments were carried out with a Micromeritics Gemini surface area analyser, stored inside a glove box under inert atmosphere and equipped with a LN Dewar with reduced volume (0.6 l). Therefore, under these conditions only a limited number of data points in the adsorption isotherm were collected and the pore size analysis through the BJH model was not feasible with reasonable accuracy.

**Figure 9:**
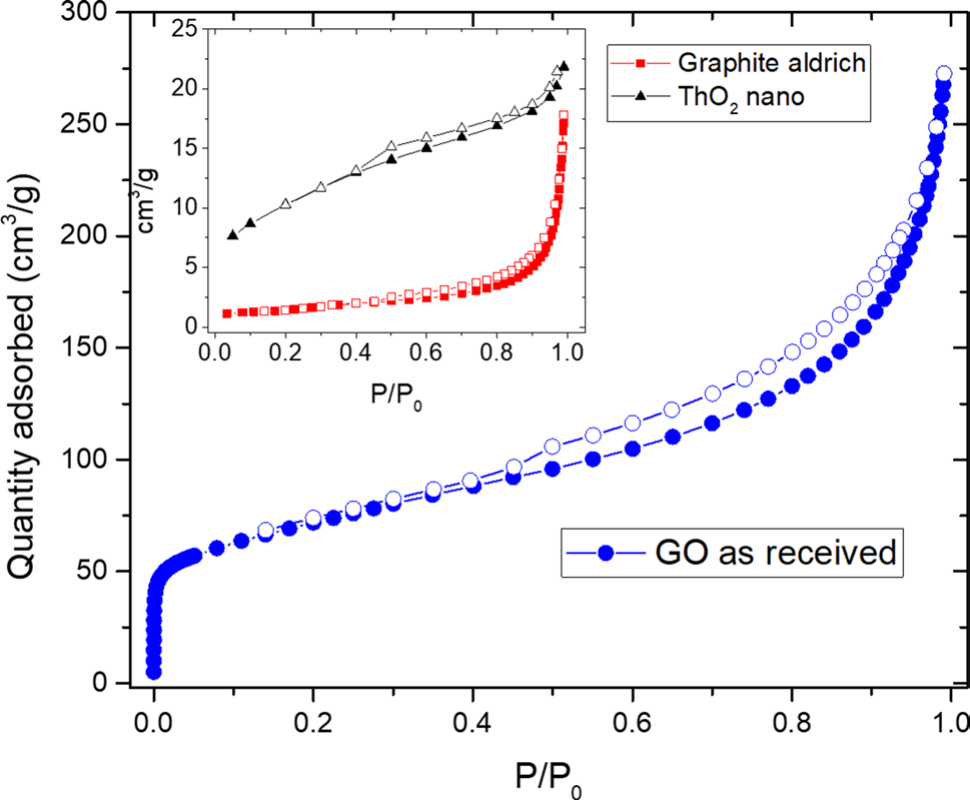
N_2_ physisorption isotherms collected from starting materials, GO, graphite and ThO_2_ nanopowders (inset graph); full symbols are for adsorption, open symbols are for desorption.

Isotherms collected for the original GO and graphite are reported in Fig. 9. GO displays a remarkably high SSA (around 250 m^2^/g) with a presence of micropores, evidenced by a steep increase in N_2_ adsorption at low relative pressures (< 10^−2^), and narrowly distributed slit-like mesopores, as indicated by the hysteresis type H3, according to IUPAC^21^. As for the pore size distribution, in the micropores range the distribution obtained using Horwath-Kawazoe model shows a maximum at 0.6 nm, whereas in the mesopores range a clear peak at around 3.8 nm is observed by applying the BJH model to desorption (pore size distribution graphs not reported).

Table 3 reports specific surface areas of the starting materials and the final carbides, evaluated using BET and single point adsorption methods.

**Table 3:** Relevant parameters obtained by N_2_ physisorption measurements on starting materials and pellets after carbothermal reduction.

Sample (set-up)	Sample mass (g)	SSA BET (m^2^/g)	SSA single point (m^2^/g)^a^	Total pore volume (cm^3^/g)^b^	Pore size (nm)^c^
nano-ThO_2_ (Gemini)	0.2455	37.9 ± 0.2	36.8 ± 0.2	0.034 ± 0.001	3.6*
Graphite (ASAP2020)	0.4424	5.2 ± 0.4	5.2 ± 0.4	0.022 ± 0.002	2.5; 3.8; 5–100
Graphene oxide (ASAP2020)	0.0706	248 ± 2	244 ± 2	0.387 ± 0.004	0.6 ; 3.8
ThCx–graphite (Gemini)	0.0949	13.4 ± 0.4	13.1 ± 0.4	0.018 ± 0.006	n.a
ThCx–GO (Gemini)	0.1076	30.7 ± 0.3	30.0 ± 0.3	0.028 ± 0.001	n.a

Uncertainty values are derived on the basis of experimental set-up used, absolute surface area and sample mass, according to Micromeritics indications (https://www.micromeritics.com/Repository/Files/micro_tech_tip_14-surface-area-analyses.pdf).

^a^Evaluated at P/P_0_ 0.3.

^b^Estimated at P/P_0_ 0.98.

^c^From a BJH desorption model for mesopores and H–K model for micropores.

*Experimental data number allows pore size evaluation.

**Figure 10:**
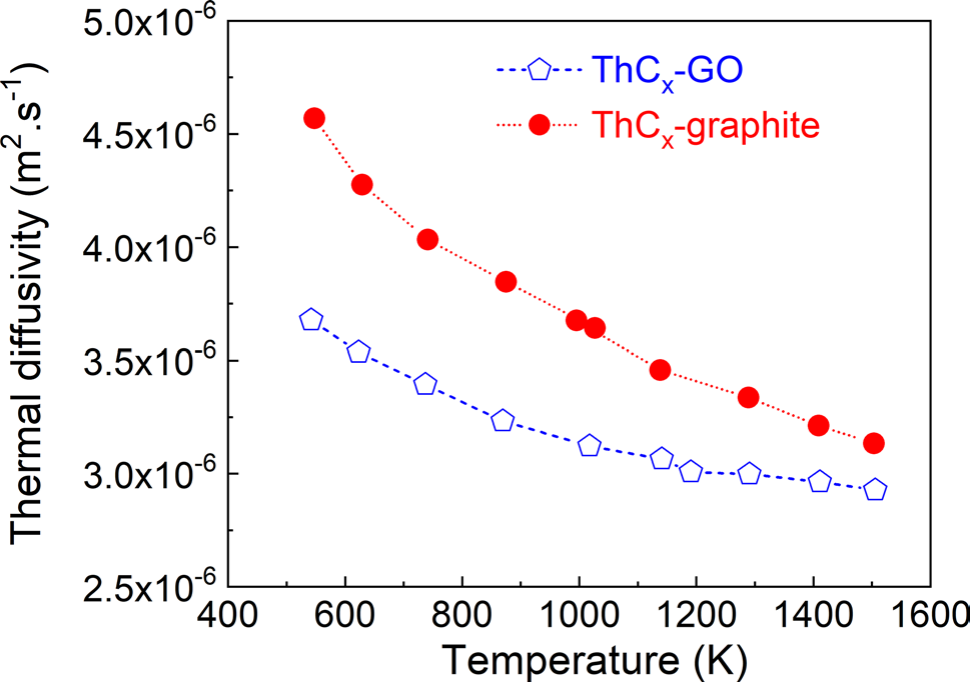
Thermal diffusivity of ThCx–GO and ThCx–graphite.

**Figure 11:**
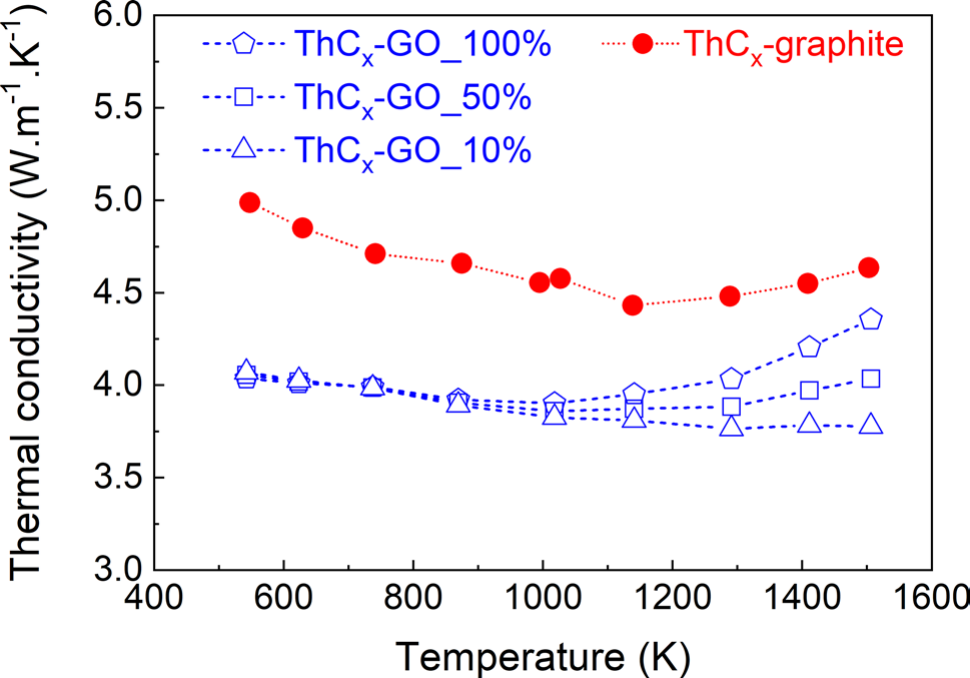
Thermal conductivity of ThCx–graphite and ThCx–GO.

Thermal diffusivity α data (in m^2^ s^−1^) measured up to 1550 K by the laser flash technique developed at JRC Karlsruhe are reported in Fig. 10. They show a higher diffusivity in the ThCx–graphite sample than in that of ThCx–GO. The difference between both samples decreases with temperature, from 28% at 500 K to 8% at 1550 K. Figure 11 shows calculations of the thermal conductivity λ obtained from the thermal diffusivity α and the density ρ (in kg m^−3^) of samples obtained after heat treatment and corrected for thermal dilatation using data from^22^, with specific heat C_p_ of ThC_2_ and ThO_2_ extracted from^23,24^ respectively. Based on XRD, ThCx–graphite is composed of only ThC_2_ within the uncertainty of the measurement, while ThCx–GO contains ThC_2_ and ThO_2_. Since the exact composition and the ThC_2_/ThO_2_ ratio of ThCx–GO is not retrievable by Rietveld analysis due to its low XRD signals and its tendency to rapidly amorphize, only rough estimates can be made. We considered three different compositions for ThCx–GO, each associated with a percentage of ThC_2_ in the sample (100% ThC_2_, 50% ThC_2_–50% ThO_2_ and 10% ThC_2_–90% ThO_2_). At low temperature, the specific heat of ThO_2_^23^ is slightly higher than that of ThC_2_^24^, whereas at high temperature the specific heat of ThC_2_ is much higher than that of ThO_2_, resulting in diverging data for the three ThCx–GO compositions. In all cases however, the ThCx–graphite conductivity is higher than that of ThCx–GO, confirming the diffusivity trends. The difference in thermal conductivity between ThCx–graphite and ThCx–GO tends to decrease with temperature. Literature about thermal conductivity data for the monoclinic α-ThC_2_ is very scarce but, as pointed out by Manara et al.^23^, an estimated value is 24 W/(m K) obtained from electrical resistivity measurements for fully dense ThC_2_ at 298 K, whereas experimental data for a 72% ρ_th_ (theroretical density) sample yield 24 W/(m K) at 443 K and 20.5 W/(m K) at 627 K. On the other hand, Pillai and Malakkal^25,26^ reported much lower values for the thermal conductivity of 94% ρ_th_ and 95% ρ_th_ ThC_2_ samples in the 300–1200 K temperature range. In particular, around 600 K the ThO_2_ thermal conductivity is found to be around 7^25^ or 8^26^ W/(m K), less than half the value reported for a 72% ρ_th_ ThC_2_^23^.

## Discussion

### Differences in reactivity between ThCx–graphite and ThCx–GO

In the prepared ThCx–GO samples, ThO_2_ is observed, in the inner of the pellets; additionally during the carboreduction less weight loss and a lower CO release are observed. All these points together can be attributed to the inefficient reduction of GO in argon atmosphere and incomplete transformation. Indeed, the literature reports that the thermal reduction of GO becomes efficient at 2023 K under high vacuum or reducing atmosphere (H_2_ or Ar–H_2_). The oxygen atoms in GO are present as hydroxyl, epoxy and carboxyl groups, of which the most thermally stable are the latter two^27^. In this study, the carbothermal reduction process was run under Argon atmosphere and up to 1923 K, so a lower efficiency seems reasonable.

The 21 wt% of graphite or GO in the starting mixtures with nano-ThO_2_ are sufficient to receive complete reduction to ThC_2_ plus the formation of carbon. The oxygen content in the starting GO (5.5 wt%) drives the reaction stoichiometry to ThO_2_ so that during the carboreduction it seems reasonable that a lower amount of carbon is formed. Since the carbothermal reduction of ThO_2_ is a solid-state diffusion process a lower amount of one component could reduce the contact and therefor affect the conversion negatively. Additionally the GO shows a tenfold higher SSA compared to graphite (Table 3); this indicates the presence of a less dense structure with lower contacts, which is reflected as well in the observed lower thermal diffusivity and conductivity of the ThCx–GO compared to ThCx–graphite. A less dense structure could be another reason why the surface controlled transformation of the nano-ThO_2_ is slower in the carbothermal reduction with GO than with graphite.

These two points together make us come to the conclusion that the ThO_2_ present in the ThCx–GO is unreacted nano-ThO_2_. In order to examine this further experiments are planned.

On the other hand, the reduced reactivity of the ThO_2_-GO mixture has the positive effect of preserving part of its initial large surface area, with a clear effect on the final material SSA and the total pore volume. A more voluminous structure of the final ThC_2_ product seems to be advantageous with respect for the irradiation and isotope yield later and makes further experiments even more attractive.

### Differences in sample behavior in air

Literature about the amorphization of thorium carbides is limited to one work^28^, which can be included in the more general research on hydrolysis of ThC_2_^29,30^. The hydrolysis of ThC_2_ in air at room temperature was found to be accompanied by the production of several different hydrocarbon gases and to result in the formation of ThO_2_ and an amorphous phase, which was not fully identified but thought to be hydrated ThO_2_. The effect of exposing UC_x_–graphite to moist air at 30 °C for 48 h was investigated in a previous work^31^. Despite a visible (but not quantifiable) carbon depletion and oxygen enrichment on the surface, the morphology of carbide grains remained unaltered, testifying to the great stability of the carbide–graphite system to external conditions.

The ThCx–GO phase evolution during XRD investigations (Fig. 4) seems to be in agreement with this phenomenon, whereas ThCx–graphite was found to be stable throughout the entire measurements campaign; ThCx–GO is more reactive towards air than ThCx–graphite. This different reactivity against air could be explained as well by a more voluminous structure of ThCx–GO compared to ThCx–graphite facilitating its reaction with air.

Based on the findings of this work we present the synthesis of ThC_2_ (ThCx–graphite) as product of the carboreduction of nano-ThO_2_ with graphite as suitable starting materials for the production of radioisotopes. The carboreduction of nano-ThO_2_ with graphene oxide forms ThCx–GO, a product of incomplete carboreduction and a mixture of ThO_2_, carbon and ThC_2_. However, properties like the lower thermal diffusivity and conductivity or the higher SSA of the ThCx–GO compared to ThCx–graphite support a voluminous structure being attractive for the recovery of radioisotopes after irradiation. This makes the nano-ThO_2_ carboreduction with GO becoming worth for further investigations towards the development of target materials for irradiation experiments dedicated to isotope production for medicinal applications.

## Methods

### Samples preparation

The process going from sample preparation to pressing of the pellet took place in glove box equipped with an MBraun gas purification system. The samples were kept under nitrogen atmosphere with a content of H_2_O < 1 ppm and of O_2_ < 50 ppm. ThO_2_ nanoparticle sample was prepared following a method described in the literature^15^. A Th(IV) solution was prepared by dissolving Th(NO_3_)_4_·5H_2_O in demineralized water. Then a precipitation was induced by adding 2.2 mol equivalents of 1 M solution of oxalic acid at 333 K under constant stirring. The resulting precipitate was washed with water and centrifuged at 2000 rpm for 5 min. The process was repeated three times. Afterwards, working under argon, the autoclave was filled with about 5.5 g oxalate powder and 3 ml of water and the hydrothermal reaction was performed for 18 h at 523 K. The resulting ThO_2_ nanocrystal’s were washed with water, ethanol and acetone, in order to gradually decrease the polarity of the solution. Each washing step was followed by centrifugation and the supernatant was removed, the residue was dried by evaporating the acetone from the last washing. Both graphite and graphene oxide were used as carbon sources for the synthesis of ThC_2_. Graphite powders of size < 45 μm and graphene oxide powder (15–20 layers, 4–10% edge oxidized) were purchased from Sigma-Aldrich. The reactants were first individually mixed in a planetary ball-mill (2 h at 3000 rpm), with jar and balls made of ZrO_2_, leading to a powder size < 5–10 μm. The powders were then mixed in an agate mortar using the mass percentages reported in Table 1, corresponding to the planned reaction.

1 $$ {\text{ThO}}_{{2}} + {\text{ 6C}} \to {\text{ThC}}_{{2}} + {\text{ 2C }} + {\text{ 2CO}}. $$

A solution of 2 wt% of phenolic resin in acetone (15 wt%) was added dropwise as binder. The powders were then further mixed in the ball mill (3 h at 3000 rpm). The pressing conditions and the heat treatment conditions were similar to those reported in^17^. At least five pellets of 13 mm diameter and about 1 mm thickness were prepared for each batch (ThO_2_–Graphite and ThO_2_–GO). The heat treatment of the samples was done by batch with a Degussa Type VSL annealing furnace under Argon flow (5 N) at a flow rate estimated 3–5 l/min. Before flushing with Ar passing through a moisture trap, we first proceed with a primary vacuum of 10^–3^ mbar of the furnace chamber. During the heat treatment, a slight Ar overpressure in the furnace is present. The furnace is located in a glove box under N_2_ whose oxygen trace is below 0.5%. Leakage into the furnace before Ar flushing cannot be excluded. However, it is limited to the trace of oxygen present in the glove box whose content is below 0.5%. The samples were maintained on a graphite crucible standing on alumina disk deposited on molybdenum grids at 1963 K for 24 h, heating rate 2 K/min. To follow the process of the carbothermal reaction, monitoring of the CO evolution during the heat treatment, we used a Residual Gas Analyser (Siemens Ultramat 6). With an analytical balance Mettler Toledo Model SAG204, we determined the weight loss after thermal treatment of the samples.

### Samples chemical and physical characterization

ELTRA CS-800 instrument was used to analyse the carbon and oxygen contents on powdered samples. The method goes through direct combustion of samples using the infrared absorption detection technique^17^. The bulk density ρ_bulk_ was measured via the weight over volume ratio; the total porosity of the samples was calculated by: P_tot_ = 1 − ρ_bulk_/ρ_th_, where ρ_th_ is the theoretical density of the final sample, calculated by the mixture rule taking into account the volumetric fractions of ThC_2_ and free carbon present in the final samples (ThC_2_ + 2C) and their theoretical solid densities, 9.14 g/cm^3^ for ThC_2_^23^, between 1.9 and 2.3 g/cm^3^ for carbon^19^, averaging the obtained results. The shrinkage percentage was determined considering only the average thickness of the pellet measured with an absolute Digimatic Indicator ID-S (Mitutoyo), before and after heat treatment. Scanning electron microscopy was performed on a Philips XL 40 using a tungsten filament (200 V–30 keV).The Transmission Electron Microscope (TEM) study was performed using an FEI Tecnai G2 model, equipped with a GATAN Tridiem camera and a GATAN Imaging Filter. The field emission gun was operated at 200 kV during the study. The TEM was adapted for the examination of highly active or irradiated nuclear materials thanks to a flange that was inserted in the octagon and which hosts the objective lenses and a glove box mounted on this flange around the CompuStage rotation holder^32^. The sample was prepared by crushing, as explained in previous work^33^; the so-prepared sample grids were then introduced into a Plasma cleaner machine in order to eliminate any organic residues, and brought to the TEM for the analysis making use of a La Calhène DPTE system. Samples embedded in oil were characterized by powder X-ray diffraction on a Rigaku MiniFlex 600 diffractometer in θ–2θ configuration using Cu K_α1–α2_ radiation. The patterns were measured from 5° to 120° in 2Θ angle with a scan/step of 0.02° scan/step and a scan speed of 0.29°/min. The software Topas version 5 was used to refine the XRD data.

### Functional properties characterization

A shielded “laser-flash” device designed and constructed at JRC Karlsruhe^34,35^ was used for the measurement of the thermal diffusivity α of the samples. The detailed description has been provided in previous study on UC_2_ samples^17^. The precision of measurements is better than 5% and is mainly determined by the variation in sample thickness. The experiments were carried out starting at about 500 K with the aim of measuring α up to the maximum temperature of about 1550 K and then back to 500 K in order to observe the effect of possible recovery effects or sample composition changes (oxidation) that might have taken place during laboratory thermal annealing.

## Supplementary Information

Supplementary Information.

## Data Availability

The datasets generated and/or analysed during the current study are available from the corresponding author on reasonable request.

## References

[1] Kim YS (2012). An overview of targeted alpha therapy. Tumor Biol..

[2] Robertson AKH (2018). Development of 225Ac radiopharmaceuticals: TRIUMF perspectives and experiences. Curr. Radiopharm..

[3] Griswold JR (2016). Large scale accelerator production of 225Ac: Effective cross sections for 78–192 MeV protons incident on 232Th targets. Appl. Radiat. Isot..

[4] Melville G (2009). Cyclotron and linac production of Ac-225. Appl. Radiat. Isot..

[5] Robertson AKH (2019). Design of a thorium metal target for 225Ac production at TRIUMF. Instruments.

[6] Formento Cavaier R (2017). Terbium radionuclides for theranostics applications: A focus on MEDICIS-PROMED. Phys. Procedia.

[7] Hoehr C (2017). Medical isotope production at TRIUMF—From imaging to treatment. Phys. Procedia..

[8] Borgna F (2017). A preliminary study for the production of high specific activity radionuclides for nuclear medicine obtained with the isotope separation on line technique. Appl. Radiat. Isot..

[9] Popescu L (2014). Nuclear-physics applications of MYRRHA. EPJ Web Conf..

[10] Ramos JP (2020). Thick solid targets for the production and online release of radioisotopes: The importance of the material characteristics—A review. Nucl. Instrum. Methods Phys. Res. B.

[11] Evensen AHM (1997). Release and yields from thorium and uranium targets irradiated with a pulsed proton beam. Nucl. Instrum. Methods Phys. Res. B.

[12] Corradetti S (2020). Nanocrystalline titanium carbide/carbon composites as irradiation targets for isotopes production. Ceram. Int..

[13] Ramos JP (2016). Target nanomaterials at CERN-ISOLDE: Synthesis and release data. Nucl. Inst. Methods Phys. Res. B.

[14] Walter O (2016). Hydrothermal decomposition of actinide (IV) oxalates: A new aqueous route towards reactive actinide oxide nanocrystals. Open Chem..

[15] Balice L (2018). Nano and micro U1-xThxO2 solid solutions: From powders to pellets. J. Nucl. Mater..

[16] Silvain J-F (2020). A review of processing of Cu/C base plate composites for interfacial control and improved properties. Int. J. Extrem. Manuf..

[17] Biasetto L (2018). Morphological and functional effects of graphene on the synthesis of uranium carbide for isotopes production targets. Sci. Rep..

[18] Becerril HA (2008). Evaluation of solution-processed reduced graphene oxide films as transparent conductors. ACS Nano.

[19] Corradetti S (2017). Graphene derived lanthanum carbide targets for the SPES ISOL facility. Ceram. Int..

[20] Alam SN (2017). Synthesis of graphene oxide (GO) by modified hummers method and its thermal reduction to obtain reduced graphene oxide (rGO). Graphene.

[21] Sing KSW (1985). Reporting physisorption data for gas/solid systems with special reference to the determination of surface area and porosity (Recommendations 1984). Pure Appl. Chem..

[22] Holleck H (1986). Material selection for hard coatings. J. Vac. Sci. Technol., A.

[23] Manara D, De Bruycker F, Sengupta AK, Agarwal R, Kamath HS, Konings RJM (2012). Thermodynamic and thermophysical properties of the actinide carbides. Comprehensive Nuclear Materials.

[24] Konings RJM (2014). The thermodynamic properties of the f-elements and their compounds. Part 2. The lanthanide and actinide oxides. J. Phys. Chem. Ref. Data.

[25] Pillai CGS (2000). Thermal conductivity of ThO2 and Th0.98U0.02O2. J. Nucl. Mater..

[26] Malakkal L (2019). Thermal conductivity of bulk and porous ThO2: Atomistic and experimental study. J. Alloy. Compd..

[27] Pei S, Cheng H-M (2012). The reduction of graphene oxide. Carbon.

[28] Lawrance JJ, O’Connor DJ (1962). Hydrolysis of thorium carbides. J. Nucl. Mater..

[29] Griess JC (1974). Hydrolysis of Lanthanide and Actinide Carbides: A Survey of Recent Literature Report ORNL-TM—4489.

[30] Bradley MJ, Ferris LM (1965). Hydrolysis of thorium carbides between 25 and 99° C. J. Inorg. Nucl. Chem..

[31] Biasetto L (2010). Developing uranium dicarbide–graphite porous materials for the SPES project. J. Nucl. Mater..

[32] Wiss T (2013). Recent results of microstructural characterization of irradiated light water reactor fuels using scanning and transmission electron microscopy. JOM.

[33] Wiss T (2015). TEM study of alpha-damaged plutonium and americium dioxides. J. Mater. Res..

[34] Sheindlin M (1998). Advances in the use of laser-flash techniques for thermal diffusivity measurement. Rev. Sci. Instrum..

[35] Staicu D (2014). Effect of burn-up on the thermal conductivity of uranium–gadolinium dioxide up to 100 GWd/tHM. J. Nucl. Mater..

