# Supplementary figures and images for: Effect of graphite and graphene oxide on thorium carbide microstructural and thermal properties
Source: Sci Rep. 2021 Apr 27;11:9058. doi: 10.1038/s41598-021-87621-0 (PMC8079694; doi:10.1038/s41598-021-87621-0)

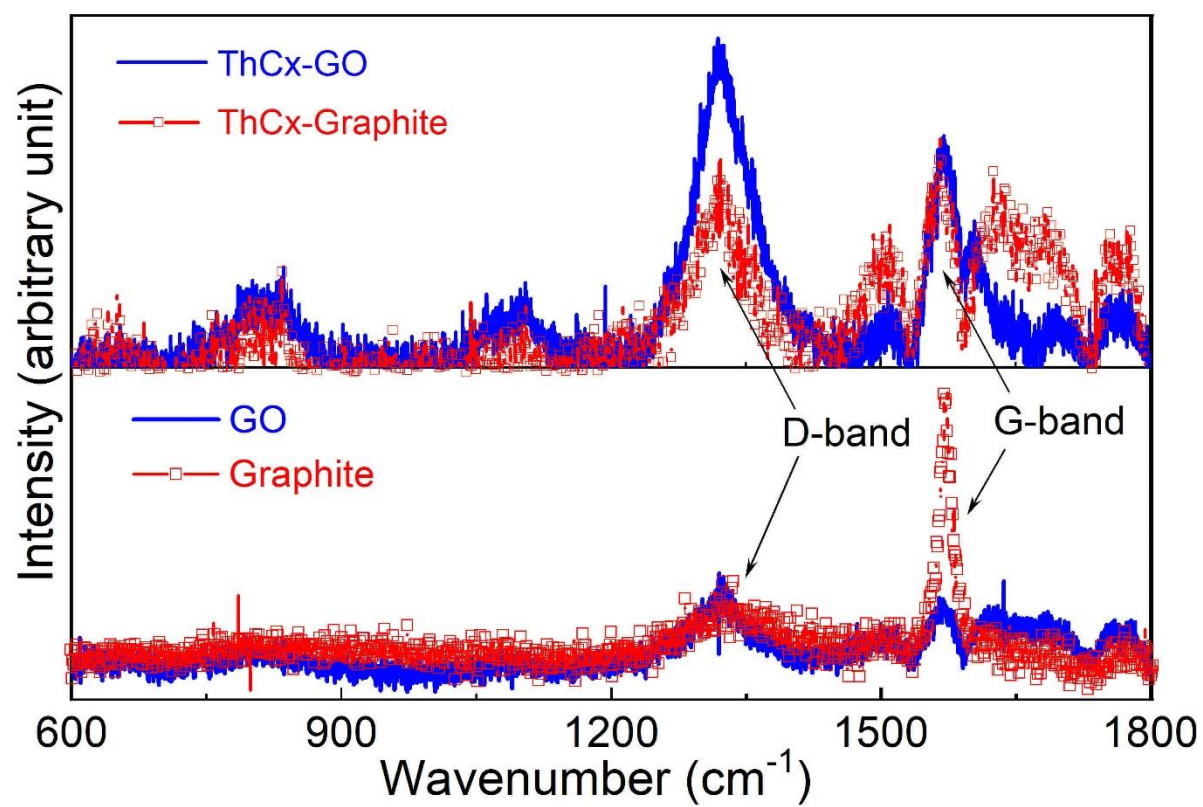

Raman spectra of graphite, GO, ThCx-graphite and ThCx-GO samples.

Supplement: Supplementary file 1 — Supplementary Information. [file 41598_2021_87621_MOESM1_ESM.pdf]
